# Medical Education and State Medical Examinations

**Published:** 1897-11

**Authors:** W. D. Hamaker

**Affiliations:** Meadville, Pa., Member of the Pennsylvania State Board of Medical Examiners


### MEDICAL EDUCATION AND STATE MEDICAL EXAMINATIONS.

CONDUCTED BY WILLIAM WARREN POTTER, M. D., Buffalo, N. Y.,
Member New York State Medical Examining and Licensing Board.

### SOME GEMS FROM THE RECENT EXAMINATION PAPERS.

BY W. D. HAMAKER, M. D., Meadville, Pa.,
Member of the Pennsylvania State Board of Medical Examiners.

THERE still remain a few people in Pennsylvania who think that an examining board is unnecessary, and there are a number in the medical profession who think that the examinations should be very easy and that no special stress should be laid on preliminary education. In order that the profession may know some of the ridiculous answers to questions and some of the evidences of lack of elementary education, I desire to give the readers of the *Pennsylvania Medical Journal* a list of some of the answers that I picked up in the recent examination held in June.

One applicant spelled tongue, "tong"; another spelled salts, "saults"; another spelled broom, "brougham"; another spelled ceratum, "serratim"; another spelled fly, "fley," and flies, "flyes." One young lady spelled sugar, "shugar"; another applicant spelled Spain, "Spane." Fir-tree was spelled "fur-tree"; another bright young man had a new way to spell rochelle, and spelled it "roachsheel," and another spelled pulse "pultz"; and sore was spelled "soar"; another spelled pus, "puss." "Corpustle," "pacients," and "beene" were found. In addition to the bright spelling quoted above, great lapses were found in grammar.

Some answers to the questions were quite amusing. The following are some of the most striking : "Hydrogen gass is degenerated from the urea." "Cantharides is derived from the root of the plant." "Pix liquida is from the Pinus Somniferous group." "Cantharides is derived from the destructive distillation of the Spanish fly." "The malar bone articulates with the occipital bone." "Picrotoxin is an alkaloid of senna and rhubarb." "Spartein and eserine are alkaloids of somnis papaverum." One said that "Spartein was derived from Sparta," and another said it "was derived from Spartus." Another said "that vinegar was an antidote for mineral acid poisons," and another stated that "an infusion of Spanish fly was one of the official preparations." Another gave us the information that "the uriniferous tubules secrete the seminal fluid." Another said that "belladonna locks up all of the secretions except the urine and feces." Another stated, without additional matter of any kind, that "the differential diagnosis between epilepsy and hysteria is that in epilepsy they fall on the stove and burn themselves and in hysteria they don't." Turning to obstetrics we found one man who, in rigid os, "would decapitate or perform craniotomy or would put on the forceps and deliver at once." Another in performing version, "would put his finger in the child's mouth and bring the chin under the os pubis and hold his hand over the mouth to prevent the liquor amnia from choking it." Another stated "that the endocardium is a mucous membrane which weighs two ounces, and is separated from the pleura by the pericardium." Another stated "that the function of the optic nerve is to contract the pupil and move the eyeball." A new diagnostic symptom was offered by another man whose paper stated "that in cerebral hemorrhage the patient may vomit the cerebro-spinal fluid!" And yet all these men have diplomas.—*Pennsylvania Medical Journal.*

---

### A NEW LAW GOVERNING TRANSIENT PRACTITIONERS OF MEDICINE.

THE following amendment of an act (*Pennsylvania Med. Jour.*, August, 1897,) to prevent incompetent persons from engaging in the practice of medicine was passed at the last session of the legislature of this state and was approved by Governor Hastings:

AN ACT

To amend the fourth section of an act, entitled An act to protect the people of the commonwealth against incompetent practitioners of medicine, surgery and obstetrics, approved the twenty-fourth day of March, one thousand eight hundred and seventy-seven, so as to require a license to be taken out by transient practitioners, who practise medicine gratuitously or so advertise, as well as those who practise for a valuable consideration.

Section 1. Be it enacted, etc., that section four of an act, entitled An act to protect the people of the commonwealth against incompetent practitioners of medicine, surgery and obstetrics, approved the twenty-fourth day of March, one thousand eight hundred and seventy-seven, which reads as follows :

Section 4. Any person who shall attempt to practise medicine or surgery for a valuable consideration, by opening a transient office within this commonwealth, or who shall, by handbills or other form of written or printed advertisement, assign such transient office or other place to persons seeking medicine or surgical advice or prescription, or who shall itinerate from place to place or from house to house, and shall propose to cure any person sick or afflicted by the use of any medicine, means or agency whatsoever for a valuable consideration, shall, before being allowed to practise in this manner, appear before the clerk of the court of quarter sessions of the county wherein such person desires to practise and shall furnish satisfactory evidence to such clerk that the provisions of this act have been complied with, and shall in addition take out a license for one year, and pay into the county treasury for the use of such county the sum of fifty dollars therefor ; whereupon it shall be the duty of such clerk to issue to such applicant a proper certificate of license on payment of the fee of five dollars for his services, be and the same is hereby amended so as to read as follows : Section 4. Any person who shall attempt to practise medicine or surgery either for a valuable consideration or without any charge or remuneration therefor, by opening a transient office within this commonwealth, or who shall, by handbill or other form of written or printed advertisements, assign such transient office or other place to persons seeking medicine or surgical advice or prescription, or who shall itinerate from place to place or from house to house, and shall propose to cure any person sick or afflicted by the use of any medicine, means or agency whatsoever, either for a valuable consideration or without any charge or remuneration therefor, shall, before being allowed to practise in this manner, appear before the clerk of the court of quarter sessions of the county wherein such person desires to practise, and shall furnish satisfactory evidence to such clerk that the provisions of this act have been complied with, and shall, in addition, take out a license for each day, and pay into the county treasury for the use of such county the sum of ten dollars therefor ; whereupon it shall be the duty of such clerk to issue to such applicant a proper certificate of license on payment of the fee of five dollars for his services.

Section 2. All acts or parts of acts inconsistent herewith are hereby repealed.

It is not clear to us just what it is intended to accomplish by this amendment. While the act apparently countenances the practice of medicine by traveling and advertising doctors it would seem that the imposition of a license fee of $10 for each day would so discourage these irregulars as to practically drive them out of the state. If, as it would seem on its face, it will subject all advertisers to the payment of a daily fee of $10 it may serve a good purpose, but that such practitioners should be legalised at any price seems questionable, to say the least in its favor.

---

### THE WISCONSIN LAW.

There are some defects in the new medical practice act of Wisconsin (*Medical Standard*, September, 1897,) which should not appear in a law passed at this time by the legislature of so progressive a state as Wisconsin.

These shortcomings, however, are mostly duplications of mistakes made by other states.

A feature of the Wisconsin law which is common to nearly all laws of this class—medical, pharmacal, dental, etc.,—is the provision requiring that the governor shall appoint the membership of the board of examiners from a list submitted by certain state medical associations. The incorporation of this provision, in a mandatory form, in the law would appear to indicate that the lawyers of the legislature gave the measure but slight attention, since it is well established in constitutional rulings that such a provision is not only of no legal force, but furnishes a basis for suits to test the validity of the law. Any law which restricts the chief executive in the free exercise of the appointing power will be declared unconstitutional when brought before the proper tribunal.

The latest decision to establish this fact clearly is that of the Supreme Court of Minnesota in the case of the state pharmaceutical association against the governor, who ignored the recommendations of the association and appointed a gentleman whose name was not on the list submitted. While this provision, doubtless, would be held by the same authority as not invalidating any act of the board in the performance of its duties, it would have to be considered were the entire act in all its features brought into question by some one opposed to its execution.

To avoid the possibility of legal complications, the appointing clause in medical practice acts should read substantially as follows:

> The governor shall appoint, with the advice and consent of the senate, a board to be known as the state board of medical examiners, to consist of . . physicians legally registered in the state of . . . to be chosen as nearly as may be from the regular, homeopathic, eclectic or other systems of medical practice, in such manner as shall approximate in the number of members accredited to each system the numerical proportion of the physicians professing such systems, respectively, in the state. The state medical societies representing the various systems of medical practice, respectively, may, upon the occurrence of a vacancy or vacancies in the representation on said board of medical examiners, report to the governor direct, recommending the names of at least three persons for every such vacancy.

There is no provision in the Wisconsin law for re-registration. For this, it may be said, the state would have had but few precedents. With the opportunity for observation furnished by the experience of their own and other states, however, this requirement should have been recognised by the promoters of the law as an indispensable feature. The simplest and most effective plan for keeping out unqualified practitioners is by annual re-registration. There is no other way of securing absolutely reliable statistics and of determining that the law is uniformly enforced. The re-registration clause will be an important part of the laws of the future.

The law makes the secretary a member of the board. This has been found in the experience of other states an undesirable provision. The secretary is the executive officer of the board, and in the discharge of such duties should not serve in the capacity of a member of the board. The salary allowed the secretary, of $300, is wholly inadequate and affords no just compensation for the actual time and labor required for the discharge of the duties of the position.

---

### NEW YORK STATE DENTAL EXAMINERS.

The annual meeting of the New York State Board of Dental Examiners was held in Albany, October 2, 1897. The relation of the New York examiners, the New York dentists and New York colleges to those of other states was carefully considered. It was decided that the methods and ends sought by the National Association of Dental Examiners were not in harmony with those of this state and the board would not, therefore, join that association or act with it. Only once had it been represented in the national association, and then it was soon discovered that the body had no recognised standard of its own and that it was not working on the same lines as those of the New York State Board. Hence it could not continue membership in that association.

It was decided that the dental colleges of the State of New York must be protected in their higher standard. The National

Association of Dental Faculties this year materially depressed its preliminary requirements and the colleges belonging to that association will admit students which the New York schools cannot receive. It was fully determined that such measures as would effectually exclude the graduates of colleges with a low standard of preliminary acquirements should be adopted, and Drs. William Carr and William Jarvie were appointed a committee to consult with the regents and the colleges and determine what should be done, they to report at the next meeting of the board.

Dr. A. P. Southwick was elected president of the board, Dr. Frank French, secretary, and Dr. William Carr, editor of the question papers.—*Dental Practitioner*, October.

---

### THREE YEARS' COLLEGES.

WHAT can a sensible student be thinking about who will deliberately enter a college which demands only three years' course for graduation ? Does he not know that he would be barred from entering a large number of states now with a diploma from such a school, and that many more states have passed laws that will require a diploma from a four years' school after 1898 ? And yet these three-year schools seem to flourish.— *Western Medical Review.*

---

### THE GOVERNOR SUSTAINS THE MAJESTY OF THE MEDICAL LAW IN OHIO.

THE *Columbus Medical Journal*, August 17, 1897, says : In the cases of W. A. France and W. F. Hale, both of whom made an appeal to the Governor and Attorney-General from the action of the State Board of Registration and Examination in refusing them a certificate, decisions were rendered by the Governor and Attorney-General on August 4, 1897. The action of the board was sustained by these officials in their decision.

"Dr." W. A. France applied for registration, on the basis of having been a legal practitioner of medicine in the State of Ohio, on the 27th day of February, 1896. Charges were preferred against him, alleging that he had been guilty of felony and gross immorality, and that he was not, during the ten years previous to February 27, 1896, a person of good moral character. These charges were heard before the board and the application of Dr. France was rejected and a certificate refused to him on the above-named grounds. The appeal was taken to the Governor and Attorney-

General from the action of the board with the above-mentioned result.

In the case of W. F. Hale, of Jackson, Ohio, upon application for registration as legal practitioner, charges were filed alleging that W. F. Hale was not a person of good moral character during the ten years previous to February 27, 1896, and that he was guilty of gross immorality, in that he abducted the prosecuting witness in a case of abortion pending against him in the Court of Common Pleas of Jackson county. "Dr." Hale was also given a hearing before the board, and was refused a certificate. He took an appeal to the Governor and Attorney-General with the result of the Governor sustaining the board's decision in his case. It is refreshing as well as encouraging to the medical profession to have a Governor as well as an Attorney-General who have the stamina and good judgment to give their support to the advancement of the medical and surgical sciences and to the suppression of any form of quackery.

---

### ILLEGAL PRACTITIONERS.

UNLICENSED and illegal practitioners of medicine in the state of Maryland are not wanted (editorial in *Maryland Med. Jour.*) and all good, loyal physicians should help to purge the state of such undesirable material and keep out those driven from other states.

The Board of Medical examiners, whose duty it is primarily to examine candidates and license such as are fit to practise, has always kept an eye on those practising illegally and has always done its duty, but it must be remembered that the board cannot be over the whole state and, therefore, physicians themselves should take sufficient interest in the welfare of the profession in Maryland and take steps to have all quacks and persons practising, not in accordance with the laws, fined, imprisoned and driven out.

Someone must procure evidence and prosecute and if any physician knows of cases deserving notice and will communicate with the board through Dr. J. McPherson Scott, at Hagerstown, he will receive help and instruction how to act. Physicians, as well as pretenders, would do well to note the accompanying instance sent to the *Journal* and see that the board is ever active and watchful to do its full duty:

An itinerant vender of drugs, who increased the sale of his nostrums by pretending to be a physician registered in Baltimore, was brought to grief not long since in Caroline county. Upon information having been given the board of medical examiners by a physician of a neighboring county, a warrant was issued for his arrest as an illegal practitioner. He was tried in Denton and on the witness stand admitted that he was not a graduate of any medical college, neither was he a registered physician of Baltimore or elsewhere. His conviction promptly followed with fine and costs.

This incident only shows how easily the medical law could be enforced and the community be rid of quacks and unprofessionals. Physicians are the men of all others interested in the suppression of the pretender. The identity of the individual and knowledge of the facts necessary for prosecution come to them without search and with this information properly used some of the good results contemplated by the medical law would be quickly secured. There are numerous violators of the law going "unwhipt of justice" and the Caroline county case ought to have many imitators.

---

### THE ILLINOIS STATE BOARD OF HEALTH RAISES THE STANDARD OF MEDICAL EDUCATION.

At a meeting of the Illinois State Board of Health, held August 26th, at East St. Louis, (*Tri-State Med. Jour.*, September, 1897,) it was decided to raise the standard of medical education of Illinois from three years to an obligatory course of four years. Moreover, in order to matriculate at a medical college in Illinois, it will henceforth be necessary for students to pass an examination in all of the ordinary studies, algebra, as far as quadratics, elementary physics and Latin equivalent to one year's study in the high school. A presentation of a college degree in letters or science, a diploma or certificate of graduation from an academy or high school, or a certificate showing that the candidate has passed the entrance examination to an accredited college or scientific school, will also enable the applicant to enter a college.

The members present at the meeting were Louis Adelsberger, of Waterloo, president; C. B. Johnson, of Champaign; Florence W. Hunt, of Chicago; P. H. Wessell, of Moline; M. Meyerovitz, of Chicago; Z. D. French, of Lawrenceville; Julius Kohl, of Belleville and J. A. Egan, secretary.

---

### MEDICAL REGISTRATION.

During the past two years (*Med. Standard*, September, 1897,) the daily press of the West has been filled with comments on the enforcement of the laws requiring the registration of physicians in those states which adopted new medical practice acts. Missouri, Ohio and Indiana have been particularly conspicuous in this respect. The extent of this comment usually may be taken as a fair criterion of the necessity existing for the inauguration of a more strict law and is ample justification of the action of the medical profession in demanding such legislation. All classes of irresponsible practitioners do not hesitate to rush into the public prints and to make threats of employing legal processes to prevent the enforcement of the law. Such men know they have nothing to lose and if their game of bluff can be worked successfully they have gained so much. But it is a cause of regret, as also a matter of surprise, that in every state are to be found reputable physicians, entitled to registration, who for purely sentimental reasons oppose the enforcement of the law and whose interviews are ever ready for the too willing representatives of journals, the highest ideals of which are the dissemination of the sensational. How such members of the profession reconcile their acts in opposing the law with the sentiment which leads them to refuse it their compliance and support is more than can be discerned by the ordinary mind. A little thought would show that they were furnishing the real basis for the opposition to proper restrictions upon which the army of quacks raise their noisy cry. Medical laws are not made in the interest of any part of the medical profession, but have their origin in the protection of the public against the practitioner without qualification for his duties. No reputable member of the profession should seek to obstruct the operation of such a law or to render indirect aid to those who make no pretense to the claims of legitimate practitioners. The papers of Indiana are filled with threats against the law and abuse of the members of the board of medical examiners. Charges of personal spite are even made, but in no case has there appeared to be the least justification for criticism of the course of the board. This has been the history of every other board and in each case it has been equally true that those without any standing in justice before the law have resorted to personal vilification to create public sentiment in their behalf. There has been enough of this for members of the medical profession to understand the tactics fully and any sympathy or encouragement offered these persons is direct opposition to the integrity of the profession and the welfare of the public. Medical examiners should use every precaution against an injustice to any one, but favors cannot be shown because of appeals or criticism.

### GETTING IN THEIR WORK.

In April, 1895, (*Texas Med. Jour.*, March, 1897,) the law against quacks went into effect in New York, since which time the New York County Medical Society has caused the arrest of eighty-three persons for illegal practice of medicine, and has obtained convictions in fifty-one cases. These fifty-one persons have paid $3,690 in fines, and a number of them have been sent to jail besides. The legal department of the society has a large number of cases pending, says the New York *Medical Record.* Why cannot the Texas State Medical Society have a legal department to look after these ducks?

